# Anemia and Prognosis in Heart Failure With Preserved Ejection Fraction: A Systematic Review and Meta-Analysis

**DOI:** 10.7759/cureus.108370

**Published:** 2026-05-06

**Authors:** Ahmad Mohammad, Shivam Singla, Bhavna Singla, Sumble Sindhu Mahessar, Ishtiaq Ahmad, Sunita Kumawat, Ghazanfar Ali, Ali Raza Mehdi

**Affiliations:** 1 Internal Medicine, Hurley Medical Center, Flint, USA; 2 Internal Medicine, TidalHealth Peninsula Regional, Salisbury, USA; 3 Internal Medicine, Erie County Medical Center Health Campus, Buffalo, USA; 4 Internal Medicine, Liaquat University of Medical and Health Sciences, Jamshoro, PAK; 5 Internal Medicine, University of Iowa Hospitals and Clinics, Iowa City, USA; 6 Internal Medicine, St. Francis Medical Center, Lynwood, USA; 7 Internal Medicine, Medicare Hospital, Faisalabad, PAK; 8 Internal Medicine, Services Hospital, Lahore, PAK

**Keywords:** anemia, chronic kidney disease, heart failure, hemoglobin, hfpef, meta-analysis, mortality, prognosis

## Abstract

Heart failure with preserved ejection fraction (HFpEF) is an increasingly prevalent clinical syndrome associated with substantial morbidity and mortality, while effective treatment options remain comparatively limited. Anemia is a common comorbidity in HFpEF and may be linked to adverse outcomes through impaired oxygen delivery, systemic inflammation, and neurohormonal activation. However, the magnitude and consistency of its prognostic significance remain uncertain because prior studies have differed in definitions, clinical settings, and patient characteristics. This systematic review and meta-analysis synthesized data from six studies, including one post hoc randomized trial analysis, prospective registries, and observational cohorts, to evaluate the association between anemia and all-cause mortality in HFpEF. A pooled random-effects analysis yielded very low-certainty evidence suggesting that anemia may be associated with increased all-cause mortality, although considerable between-study heterogeneity was present. Narrative synthesis suggested broadly similar directional trends across geographic regions and clinical contexts, including hospitalized and stable HFpEF populations. Sensitivity analyses showed that the direction of association was generally consistent, although the magnitude of the effect varied with the exclusion of influential studies. Overall, these findings suggest that anemia may represent a potential adverse prognostic marker in HFpEF, while highlighting the need for prospective studies and interventional trials to clarify mechanisms and determine whether targeted treatment strategies improve outcomes.

## Introduction and background

Heart failure with preserved ejection fraction now accounts for approximately half of all heart failure presentations worldwide and is associated with high rates of hospitalization, impaired functional status, and mortality [1,2]. In contrast to heart failure with reduced ejection fraction, heart failure with preserved ejection fraction (HFpEF) is a heterogeneous syndrome characterized by diastolic dysfunction, ventricular-arterial stiffening, chronotropic limitation, and substantial multimorbidity. Although therapeutic options for HFpEF have expanded in recent years, prognosis remains strongly influenced by coexisting conditions [3,4].

Anemia is among the most common comorbidities observed in HFpEF, with reported prevalence ranging from approximately 30% to 70% depending on study population, care setting, and diagnostic criteria [5]. Its pathogenesis in HFpEF is multifactorial and may include chronic inflammation, renal dysfunction, nutritional deficiency, hemodilution, and impaired erythropoietin signaling. Reduced hemoglobin levels may worsen symptoms and outcomes by impairing oxygen delivery, increasing neurohormonal activation, reducing exercise tolerance, and causing microvascular dysfunction.

The prognostic relevance of anemia is well established in HFrEF, but evidence in HFpEF has been less consistent. Individual cohort studies have reported variable associations with mortality and hospitalization, partly reflecting differences in HFpEF definitions (ejection fraction thresholds ≥40% vs. ≥50%), patient comorbidity burden, clinical setting (stable outpatient vs. hospitalized populations), follow-up duration, and analytical adjustment strategies [6,7]. A few studies in the past reported that anemia was associated with increased mortality and hospitalization in HFpEF; however, additional studies have since become available, and uncertainty remains regarding the magnitude and consistency of risk across contemporary clinical settings [2-5].

From a clinical perspective, it remains important to determine whether anemia functions primarily as a marker of disease severity, an independent prognostic factor, or both. This distinction has implications for risk stratification and the interpretation of emerging interventions targeting iron deficiency, renal pathways, or related mechanisms, although direct evidence that correcting anemia improves HFpEF outcomes remains limited [8,9].

Accordingly, the objective of this systematic review and meta-analysis was to update and quantitatively synthesize available evidence regarding the association between anemia and adverse outcomes in HFpEF. We pooled adjusted hazard ratios from eligible studies to evaluate whether anemia is independently associated with all-cause mortality and related clinical outcomes across diverse patient populations and care settings.

## Review

Materials and methods

Study Design and Reporting Standards

This systematic review and meta-analysis were conducted in accordance with the Preferred Reporting Items for Systematic Reviews and Meta-Analyses (PRISMA) 2020 statement [10]. The review methodology was specified in advance and included predefined procedures for literature searching, eligibility assessment, data extraction, risk of bias appraisal, and statistical synthesis. The primary objective was to evaluate whether anemia is independently associated with adverse clinical outcomes in patients with heart failure with preserved ejection fraction. The review protocol was not prospectively registered in the International Prospective Register of Systematic Reviews (PROSPERO); this is acknowledged as a methodological limitation.

Eligibility Criteria and PICO Specification

The research question was structured according to the PICO framework [11]. The population of interest comprised adults with a clinical diagnosis of HFpEF. The exposure group consisted of patients with anemia, while the comparator group included HFpEF patients without anemia. The primary outcome was all-cause mortality. Secondary outcomes of interest included cardiovascular mortality and heart failure hospitalization when separately reported.

Eligible studies included randomized trial post hoc analyses, prospective or retrospective cohort studies, registry investigations, community surveillance datasets, and pooled individual patient-data analyses that reported extractable HFpEF-specific results. Inclusion required a defined HFpEF population, typically based on left ventricular ejection fraction thresholds of ≥50% or, in earlier studies, ≥40%; anemia defined using World Health Organization criteria or comparable prespecified thresholds; and reporting of adjusted hazard ratios (HRs) with corresponding 95% confidence intervals for all-cause mortality or other separately reported time-to-event outcomes. Where mixed heart failure populations were included, studies were eligible only if HFpEF-specific adjusted estimates were separately extractable. Studies were excluded if they did not provide adjusted effect estimates, lacked separable HFpEF data, reported only composite endpoints without separately extractable all-cause mortality estimates, and were conference abstracts only, narrative reviews, editorials, animal studies, or non-adult populations.

Search Strategy and Study Identification

A structured electronic literature search was undertaken to identify studies evaluating the association between anemia and prognosis in HFpEF. PubMed/MEDLINE, Embase, and Web of Science were searched from database inception through the most recent search update prior to final analysis. Search strategies combined controlled vocabulary and free-text terms related to HFpEF, anemia, and prognosis.

The full PubMed search strategy was as follows: (“heart failure with preserved ejection fraction” OR HFpEF OR “preserved ejection fraction heart failure” OR “diastolic heart failure”) AND (anemia OR anaemia OR hemoglobin OR haemoglobin) AND (mortality OR prognosis OR outcomes OR hospitalization OR survival).

Equivalent syntax adapted to database indexing terms was used for Embase and Web of Science. No language restrictions were applied during the initial search stage; articles requiring translation were assessed where feasible. Reference lists of eligible studies, prior reviews, and relevant pooled analyses were also manually screened to identify additional records not captured electronically, including large collaborative datasets. Titles and abstracts were screened independently, followed by full-text review of potentially eligible reports using predefined criteria. Disagreements regarding eligibility were resolved through discussion and consensus. The study selection process is summarized in the PRISMA flow diagram.

Data Collection and Variable Extraction

Data abstraction was conducted using a standardized data collection framework. Extracted variables included study design, geographic setting, sample size, proportion of patients with anemia, HFpEF definition, anemia criteria, follow-up duration, reported all-cause mortality, and other separately reported clinical endpoints, and fully adjusted hazard ratios with 95% confidence intervals. When multiple adjusted models were presented, the estimate reflecting the greatest degree of covariate adjustment was selected for analysis. Hazard ratios were logarithmically transformed, and corresponding standard errors were derived when necessary to facilitate pooled quantitative synthesis.

Assessment of Methodological Quality

Risk of bias was evaluated according to the study design. The post hoc randomized trial analysis was assessed using the risk of bias in non-randomized studies of interventions (ROBINS-I) instrument, with emphasis on confounding, participant selection, exposure classification, missing outcome data, outcome measurement, and selective reporting [12]. Observational cohort and registry studies were appraised using the Newcastle-Ottawa scale (NOS), evaluating participant selection, comparability of exposed and non-exposed groups, and adequacy of outcome ascertainment [13]. Based on these structured tools, studies were categorized as having low, moderate, or high risk of bias.

In addition to study-level appraisal, the overall certainty of evidence for the primary outcome was assessed using the Grading of Recommendations Assessment, Development and Evaluation (GRADE) framework. Domains considered included risk of bias, inconsistency, indirectness, imprecision, and publication bias. Because the available evidence was derived largely from observational studies and demonstrated substantial between-study heterogeneity, certainty ratings were interpreted with caution.

Outcomes

The primary outcome was all-cause mortality. Secondary outcomes included cardiovascular mortality and hospitalization related to heart failure when separately reported. Whenever available, outcomes were analyzed as time-to-event variables using adjusted hazard ratios in order to preferentially incorporate estimates accounting for measured confounders within individual studies.

Statistical Analysis

Quantitative synthesis was performed using Review Manager (RevMan) version 5.4 (London, UK: Cochrane Collaboration), with supplementary calculations performed using standard random-effects methods where required. Hazard ratios for the primary outcome of all-cause mortality were pooled using the generic inverse-variance approach after logarithmic transformation of effect estimates and corresponding standard errors.

A random-effects model was prespecified because clinical and methodological heterogeneity was anticipated across studies, including differences in HFpEF diagnostic thresholds, baseline comorbidity burden, anemia definitions, care settings, and duration of follow-up. Between-study heterogeneity was assessed using Cochran’s Q statistic and quantified with the I² statistic, with values interpreted according to conventional Cochrane thresholds.

Pooled hazard ratios were reported with 95% confidence intervals. Given anticipated heterogeneity, 95% prediction intervals were also calculated for the primary outcome to estimate the range of effects that might plausibly be observed in a future comparable study setting. Forest plots were generated to visually display individual study estimates and pooled effects.

Sensitivity Analysis and Evaluation of Publication Bias

Sensitivity analyses were prespecified to evaluate the robustness of findings. These included sequential leave-one-out analyses, in which the meta-analysis was repeated after omission of each study individually, as well as subgroup or restricted analyses when clinically relevant (for example, exclusion of mixed ejection-fraction cohorts or studies judged at high risk of bias). Changes in pooled effect size and heterogeneity were examined descriptively.

Formal assessment of publication bias was not undertaken because the number of studies included in the quantitative synthesis was small. Funnel plot interpretation and statistical tests for asymmetry are considered unreliable when fewer than 10 studies are available, particularly in the presence of substantial heterogeneity.

Certainty of Evidence Assessment

The overall certainty of evidence for the primary outcome was evaluated using the Grading of Recommendations Assessment, Development and Evaluation (GRADE) framework. Evidence certainty was assessed across the domains of risk of bias, inconsistency, indirectness, imprecision, and publication bias. Because the included evidence was derived predominantly from observational studies, certainty ratings were interpreted in the context of prognostic factor research. Downgrading decisions were based on study limitations, substantial statistical heterogeneity, wide uncertainty around pooled estimates where applicable, and the inability to reliably assess small-study effects because of the limited number of included studies. The final certainty rating for each major outcome was categorized as high, moderate, low, or very low.

Results

Study Selection Process

**Figure 1:**
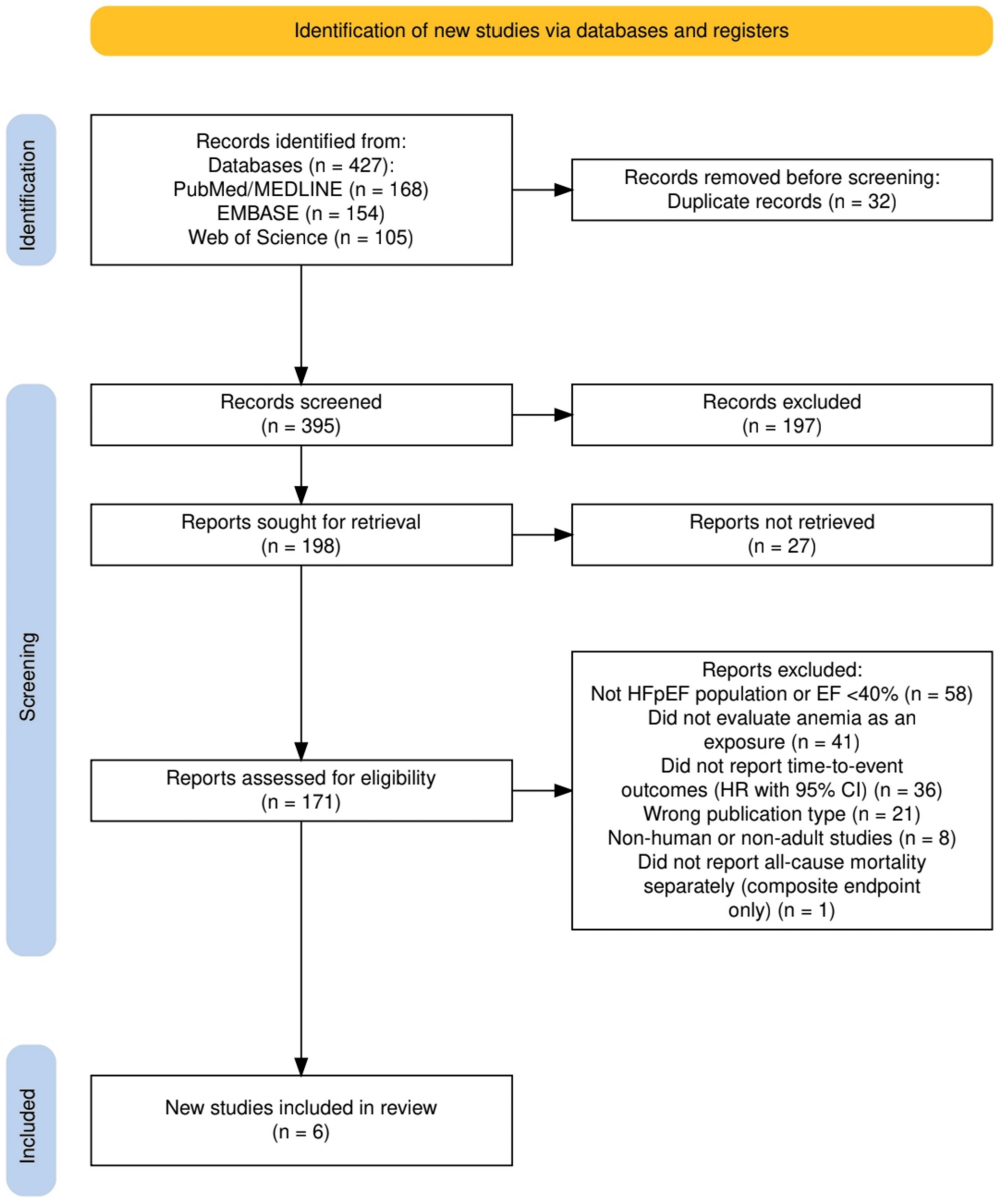
The PRISMA flowchart represents the study selection process. PRISMA: Preferred Reporting Items for Systematic Reviews and Meta-Analyses

The process of study identification and selection is depicted in Figure 1. The initial electronic search across PubMed/MEDLINE, Embase, and Web of Science yielded 427 citations. After the removal of 32 duplicate records, 395 unique records remained for preliminary screening. Title and abstract evaluation led to the exclusion of 197 records that did not meet relevance criteria. A total of 198 reports were sought for retrieval, of which 171 were successfully obtained and reviewed in detail. During eligibility assessment, studies were excluded for the following predefined reasons: inclusion of non-HFpEF populations or left ventricular ejection fraction <40% (n=58), absence of anemia as the primary exposure variable (n=41), failure to report time-to-event outcomes with adjusted hazard ratios (n=36), ineligible publication types such as narrative reviews or editorials (n=21), non-human or pediatric study populations (n=8), and failure to report all-cause mortality separately from composite endpoints (n=1). Following application of all inclusion and exclusion criteria, six studies satisfied eligibility requirements and were incorporated into both the qualitative synthesis and quantitative meta-analysis.

Characteristics of the Selected Studies

The key characteristics of the studies included in this systematic review and meta-analysis are summarized in Table 1. A total of six observational studies and post hoc trial analyses were included, encompassing diverse clinical settings and geographic regions across North America, Europe, and Asia. Sample sizes ranged from 212 to over 15,000 weighted heart failure events, with HFpEF populations defined using left ventricular ejection fraction thresholds ranging from ≥40% to ≥50%. Anemia was consistently defined using World Health Organization criteria across all included studies. Follow-up duration varied from one year to more than five years, allowing assessment of both short- and long-term outcomes. All studies reported adjusted time-to-event estimates, primarily focusing on all-cause mortality, while several also examined heart failure hospitalization, cardiovascular mortality, or related secondary outcomes. Despite heterogeneity in study design and patient populations, most studies suggested that anemia may be associated with adverse outcomes in HFpEF, although effect sizes varied across cohorts and clinical contexts.

**Table 1:** Summary of included studies evaluating the association between anemia and clinical outcomes. HFpEF: heart failure with preserved ejection fraction; Hb: hemoglobin; CKD: chronic kidney disease; HF: heart failure; CVD: cardiovascular disease; RCT: randomized controlled trial; LMIC: low- and middle-income countries; TOPCAT: Treatment of Preserved Cardiac Function Heart Failure with an Aldosterone Antagonist; NYHA: New York Heart Association; ARIC: Atherosclerosis Risk in Communities; HFmrEF: heart failure with mildly reduced ejection fraction

Studies	Design/country	Total n	Anemia (%)	HFpEF definition	Anemia definition	Follow-up	Outcomes reported	Adjusted effect (HR+95% CI)	Key adjustment variables	Notes
Gupta et al. (2020) [14]	Post hoc RCT analysis/Americas	1,748	~41	EF ≥45%	WHO (Hb <13 g/dL men, <12 g/dL women)	Median 2.4 years	Primary composite: CV death/aborted cardiac arrest/HF hospitalization; all-cause mortality; CV mortality; HF hospitalization; all-cause hospitalization	Primary composite: HR: 1.52 (1.27-1.83); all-cause mortality: HR: 1.40; CV mortality HR: 1.47; HF hospitalization HR: 1.56	Age, sex, comorbidities, renal function, treatment allocation, clinical covariates	TOPCAT-Americas HFpEF cohort; anemia prevalence corrected from source publication
Jin et al. (2019) [15]	Prospective HF registry/China	1,604	51	EF ≥50%	WHO	Median 33.9 months (IQR: 23.1-40.8)	All-cause mortality; all-cause rehospitalization	Mortality HR: 1.14 (0.85-1.52); rehospitalization HR: 1.13 (0.96-1.33)	Age, sex, NYHA class, renal function, comorbidity burden	Death incidence 17.5% vs. 10.6%; rehospitalization 44.9% vs. 37.7% (anemia vs. non-anemia)
Köseoğlu and Özlek (2024) [16]	Retrospective cohort/Turkey	212	38.2	EF ≥50%	WHO	Mean 66.2 months (~5.5 years)	All-cause mortality; iron deficiency analysis	Anemia HR: 5.401 (4.303-6.209); iron deficiency HR: 3.502 (2.204-6.701)	Multivariable clinical and laboratory covariates	Largest effect estimate; long follow-up; high risk of bias
Pintér et al. (2022) [17]	Retrospective cohort/Hungary	375	37	EF ≥40% (HFpEF+HFmrEF)	WHO	Median 1.4 years	All-cause mortality	Adjusted HR: 2.33 (1.21-4.52)	Age, sex, renal function, comorbidities	Mixed EF cohort; excluded in sensitivity analysis
Caughey et al. (2014) (ARIC) [18]	Community surveillance registry/USA	3,309 sampled cohort (15,461 weighted events descriptively)	~70-71	EF ≥40%	WHO	1 year	All-cause mortality; hospital length of stay	HFpEF HR: 2.10 (1.60-2.70)	Demographics, comorbidities, hospitalization variables	Acute hospitalized HF cohort; anemia prevalence corrected from source publication
Chairat et al. (2020) [19]	Retrospective cohort/Thailand	414 total (HFpEF n=287)	62.6 overall	EF ≥50%	WHO	1 year	All-cause mortality	HFpEF HR: 2.667 (1.216-5.853)	Age, sex, renal function, comorbidities	LMIC cohort with HFpEF subgroup

Risk of Bias Assessment

The risk of bias assessment is summarized in Table 2. Overall, most included studies demonstrated low to moderate risk of bias, reflecting the predominantly observational nature of the evidence base. The post hoc randomized trial analysis by Gupta et al. was judged to have a moderate risk of bias because anemia was an observational exposure rather than a randomized intervention, leaving potential for residual confounding [14]. Prospective registry studies generally showed stronger methodological quality, whereas retrospective cohort studies were more susceptible to selection bias and unmeasured confounding. Notably, the study by Köseoğlu and Özlek was assessed as high risk of bias because of its retrospective design, small sample size, and unusually large effect estimate, which may indicate residual confounding or cohort-specific factors [16]. Registry-based and community surveillance studies demonstrated acceptable comparability and outcome assessment, although follow-up completeness and adjustment strategies varied. Overall, the risk of bias profile supports cautious interpretation of the pooled findings.

**Table 2:** Risk of bias assessment of studies evaluating anemia and clinical outcomes. ROBINS-I: risk of bias in non-randomized studies of interventions; NOS: Newcastle-Ottawa scale; RCT: randomized controlled trial; Hb: hemoglobin; LMIC: low- and middle-income countries; RoB: risk of bias; ARIC: Atherosclerosis Risk in Communities

Studies	Design	Risk of bias tool	RoB domain summary	Overall RoB judgment
Gupta et al. (2020) [14]	Post hoc analysis of an RCT	ROBINS-I	Confounding: moderate (anemia not randomized); selection: low; classification of exposure: low; missing data: low; outcome measurement: low; reporting: low	Moderate risk
Jin et al. (2019) [15]	Prospective registry	NOS	Selection: 3/4 stars; comparability: 1/2 stars; outcome: 2/3 stars (no explicit follow-up duration)	Moderate risk
Köseoğlu and Özlek (2024) [16]	Retrospective cohort	NOS	Selection: 3/4 stars; comparability: 1/2 stars; outcome: 2/3 stars (retrospective data, very large HR suggests confounding)	High risk
Pintér et al. (2022) [17]	Retrospective cohort	NOS	Selection: 3/4 stars; comparability: 2/2 stars; outcome: 2/3 stars	Low-moderate risk
Caughey et al. (2014) (ARIC) [18]	Community surveillance registry	NOS	Selection: 3/4 stars; comparability: 2/2 stars; outcome: 2/3 stars (registry-based, weighted sample)	Low-moderate risk
Chairat et al. (2020) [19]	Retrospective cohort	NOS	Selection: 2/4 stars (LMIC hospital-based); comparability: 1/2 stars; outcome: 2/3 stars	Moderate

All-Cause Mortality

**Figure 2:**
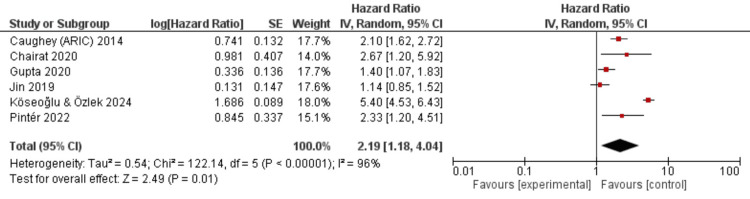
Forest plot of adjusted hazard ratios for all-cause mortality in patients with heart failure with preserved ejection fraction and anemia. ARIC: Atherosclerosis Risk in Communities

Across six included studies reporting adjusted hazard ratios for all-cause mortality, available evidence suggested that anemia may be associated with a higher risk of death in patients with heart failure with preserved ejection fraction (HFpEF). Using a random-effects model, the pooled analysis yielded a hazard ratio of 2.19 (95% CI: 1.18-4.04), indicating that anemic patients had a numerically higher estimated mortality risk than non-anemic patients, although certainty in the estimate remains limited (Figure 2). Considerable between-study heterogeneity was observed (I²=96%, τ²=0.54, p<0.00001), indicating substantial variability in effect estimates across studies. Potential contributors to heterogeneity include differences in study populations, ejection fraction thresholds, anemia severity and definitions, clinical setting (hospitalized vs. stable outpatient cohorts), comorbidity burden, and duration of follow-up. Given the small number of included studies and marked heterogeneity, the pooled estimate should be interpreted cautiously. Overall, these findings provide very low-certainty evidence that anemia may be associated with increased all-cause mortality in HFpEF, but the magnitude and consistency of this association remain uncertain.

Certainty of Evidence

Using the GRADE framework, the certainty of evidence for the association between anemia and all-cause mortality in patients with heart failure with preserved ejection fraction was judged to be very low (Table 3). Although the pooled estimate suggested a possible increase in mortality risk, certainty was downgraded because the included evidence was derived predominantly from observational studies, substantial inconsistency was present across studies (I²=96%), and the confidence interval around the pooled estimate remained relatively wide. In addition, publication bias or small-study effects could not be reliably assessed because only a limited number of studies were available for quantitative synthesis. Accordingly, confidence in the precise magnitude and generalizability of the pooled effect remains limited, and future well-designed studies may meaningfully change the current estimate.

**Table 3:** GRADE summary of findings for the primary outcome. †This participant count reflects the simple sum of included cohort sizes as reported in the primary studies and does not account for weighted event estimates reported in registry-based analyses such as Caughey et al. [18]. Certainty of evidence was assessed using the GRADE framework. Evidence was judged to be of very low certainty because the pooled estimate was derived predominantly from observational studies and demonstrated considerable heterogeneity, with additional imprecision and inability to meaningfully assess small-study effects given the limited number of included studies. GRADE: Grading of Recommendations Assessment, Development and Evaluation; HFpEF: heart failure with preserved ejection fraction

Outcome	Number of studies	Participants	Relative effect (95% CI)	Certainty of evidence	Reasons for rating
All-cause mortality in HFpEF patients with anemia vs. without anemia	6	5,640†	HR: 2.19 (1.18-4.04)	Very low	Observational study designs; serious inconsistency (I²=96%); imprecision with wide confidence intervals; inability to reliably assess publication bias because of the small number of studies; influence of one high-risk outlier study

Narrative Synthesis of Study-Level Differences

Given the substantial between-study heterogeneity, exploratory study-level comparisons were performed using aggregate study characteristics. Studies using stricter HFpEF definitions (EF ≥50%), including Jin et al. [15], Köseoğlu and Özlek [16], and Chairat et al., generally reported moderate-to-stronger associations between anemia and adverse outcomes [19]. In contrast, studies using broader ejection fraction thresholds (EF ≥40% or ≥45%), such as Gupta et al. [14], Caughey et al. (Atherosclerosis Risk in Communities {ARIC}) [18], and Pintér et al., also suggested increased risk, although effect estimates varied across cohorts [17]. These findings indicate that differences in HFpEF case definition may have contributed to heterogeneity.

The clinical setting was also an important potential source of variability. Hospitalized or acute-care cohorts, particularly the ARIC study, demonstrated stronger short-term risk estimates, whereas outpatient, registry, or more stable chronic-care populations showed more variable associations [18]. This pattern suggests that anemia may carry different prognostic implications across care settings and levels of baseline illness severity.

Differences in comorbidity burden, particularly renal dysfunction, age, and overall disease severity, may have further influenced results across studies. Follow-up duration also varied substantially, ranging from one year to more than five years, which may have affected the magnitude of observed associations. Overall, while the direction of effect was broadly consistent across most studies, the estimated magnitude of association varied considerably according to study design and population characteristics.

Sensitivity Analyses

Sensitivity analyses were performed to evaluate the robustness of the primary findings. Sequential leave-one-out analyses showed that the direction of association between anemia and increased all-cause mortality remained unchanged after exclusion of each study individually, although the magnitude of effect varied. The largest influence was observed after exclusion of the study by Köseoğlu and Özlek, which reduced the pooled hazard ratio from 2.25 to 1.73 and decreased heterogeneity from I²=95% to I²=65%, indicating that this study contributed substantially to between-study variability [16].

The outlying estimate reported by Köseoğlu and Özlek may plausibly reflect several study-specific features, including its retrospective single-center cohort design, small sample size, longer follow-up duration, potential residual confounding, and possible differences in patient selection or baseline disease severity [16]. These factors are clinically and methodologically relevant and may explain why this study yielded a markedly larger effect estimate than most other included cohorts.

By contrast, exclusion of the study by Pintér et al. did not materially alter the pooled estimate [17]. However, this leave-one-out result should be interpreted only as evidence of limited individual study influence and not as evidence against an EF-threshold subgroup effect. Study-level moderator analysis is the appropriate method for that question, and formal subgroup testing showed that EF threshold definition significantly moderated heterogeneity (Q-between p=0.046) across studies, including those by Gupta et al. [14], Pintér et al. [17], and Caughey et al. (Table 4) [18].

**Table 4:** Leave-one-out sensitivity analysis for all-cause mortality. Leave-one-out analyses were performed by sequentially excluding each study and recalculating the pooled hazard ratio using a random-effects model. The largest change was observed after exclusion of the study by Köseoğlu and Özlek, which reduced the pooled HR from 2.19 to 1.68 and reduced heterogeneity from 96% to 68%, indicating that this high-risk study materially influenced the magnitude of the pooled estimate [16]. ARIC: Atherosclerosis Risk in Communities

Omitted studies	Pooled HR	95% CI	I² (%)	Interpretation
None (primary analysis)	2.19	1.18-4.04	96	Primary random-effects model
Gupta et al. (2020) [14]	2.45	1.24-4.82	96	Direction unchanged
Jin et al. (2019) [15]	2.56	1.36-4.82	96	Direction unchanged
Köseoğlu and Özlek (2024) [16]	1.68	1.28-2.21	68	Most influential study; heterogeneity substantially reduced
Pintér et al. (2022) [17]	2.20	1.16-4.18	97	Mixed EF cohort exclusion did not materially alter result
Caughey et al. (2014) (ARIC) [18]	2.28	1.10-4.70	97	Direction unchanged
Chairat et al. (2020) [19]	2.17	1.15-4.09	97	Direction unchanged

Additional sensitivity analysis using the Hartung-Knapp-Sidik-Jonkman method yielded directionally similar results with wider confidence intervals, reflecting greater statistical uncertainty under a more conservative variance estimator (Table 5). Overall, these analyses suggest that while the pooled association was generally stable in direction, the magnitude of the effect was sensitive to influential individual studies and should therefore be interpreted with caution.

**Table 5:** Comparison of random-effects inference methods for all-cause mortality. *The Hartung-Knapp-Sidik-Jonkman (HKSJ) method was used to calculate confidence intervals as a sensitivity analysis, providing more conservative estimates given the small number of included studies and considerable between-study heterogeneity.

Model	Pooled HR	95% CI	I² (%)	95% prediction interval	Interpretation
DerSimonian-Laird random-effects model	2.19	1.18-4.04	96	0.39-12.21	Possible pooled association, but substantial heterogeneity
Hartung-Knapp-Sidik-Jonkman sensitivity analysis	2.19	1.05-4.57*	96	Not separately estimated	Directionally similar result with more conservative uncertainty bounds

Discussion

In this systematic review and meta-analysis, very low-certainty evidence suggested that anemia may be associated with a higher risk of all-cause mortality in patients with heart failure with preserved ejection fraction (HFpEF). Across diverse settings, including randomized trial cohorts, registry populations, and real-world observational studies, anemic HFpEF patients generally demonstrated higher estimated mortality risk than their non-anemic counterparts. However, the magnitude of association varied substantially across studies, and considerable between-study heterogeneity was observed. Accordingly, while the pooled estimate suggests that anemia may represent an adverse prognostic marker in HFpEF, confidence in the precise effect size remains limited, and the broad prediction interval indicates that the strength of association may differ meaningfully across comparable future settings. Importantly, sensitivity analyses showed that the overall direction of effect was generally preserved after sequential exclusion of individual studies, although pooled estimates were influenced by outlying cohorts. These findings support cautious interpretation of anemia as a potential marker of increased risk in HFpEF rather than definitive evidence of an independent causal determinant of adverse outcomes.

Our findings reinforce and extend previous observations suggesting that anemia may be associated with adverse outcomes in HFpEF, aligning with prior prognostic literature and ARIC surveillance data, while incorporating more contemporary cohorts that applied standardized HFpEF criteria [18,20]. Notably, earlier studies often combined HFpEF with reduced or mildly reduced ejection fraction subgroups, potentially limiting phenotype-specific conclusions [21]. In contrast, the majority of studies included in the present review focused on EF ≥50%, strengthening the relevance of our findings to contemporary HFpEF definitions. Unlike Jin et al., who reported a non-significant association after multivariable adjustment in the overall cohort, the broader evidence base synthesized here suggests that the prognostic signal of anemia may be more apparent in studies with larger sample sizes, longer follow-up, or greater comorbidity burden [15]. Differences in comorbidity burden, clinical setting, and case definition across studies may also have contributed to variability in risk estimates. Collectively, these comparisons support the view that anemia may represent an adverse prognostic marker in HFpEF, although the magnitude of association appears to vary across populations and clinical settings [22].

Several interrelated mechanisms may help explain why anemia could confer heightened risk in HFpEF. Reduced hemoglobin levels can limit systemic oxygen delivery, potentially exacerbating the diastolic dysfunction and impaired myocardial energetics characteristic of HFpEF. This mismatch between oxygen supply and myocardial demand may promote subclinical ischemia, increased reliance on anaerobic metabolism, and heightened sympathetic activation [23]. Additionally, HFpEF is increasingly recognized as a systemic inflammatory and microvascular disorder; chronic inflammatory signaling, endothelial dysfunction, and impaired nitric oxide bioavailability may worsen in the presence of anemia, potentially compounding myocardial stiffening and impaired relaxation. Iron deficiency, which frequently coexists with anemia, may further impair mitochondrial function and exercise capacity, thereby contributing to symptom burden and disease progression. Together, these mechanisms provide a biologically plausible framework for the observed association between anemia and adverse outcomes [24,25].

An important hypothesis-generating concept arising from our analysis is the possibility of a “hemoglobin reserve threshold” in heart failure with preserved ejection fraction - that is, a level below which reductions in hemoglobin may be less well tolerated in certain patients because of limited cardiovascular reserve. Unlike heart failure with reduced ejection fraction, HFpEF is often characterized by impaired ventricular filling, reduced diastolic reserve, chronotropic limitation, and greater dependence on adequate preload and systemic perfusion [26].

This concept was not directly tested in the present study and should not be interpreted as a defined numeric threshold or treatment target. Rather, it is an exploratory framework intended to contextualize observed heterogeneity in prognostic associations. Variability in age, renal dysfunction, frailty, inflammatory burden, and overall disease severity across cohorts suggests that physiologic reserve and comorbidity burden may modify the clinical impact of anemia. Future studies using continuous hemoglobin modeling, threshold analyses, and prospective phenotype-specific cohorts are needed to determine whether clinically meaningful hemoglobin inflection points exist in HFpEF and whether such thresholds have implications for risk stratification or treatment selection [27].

The findings of this meta-analysis support careful recognition and evaluation of anemia as a clinically relevant comorbidity in the care of heart failure with preserved ejection fraction. Given the observed association between anemia and adverse outcomes, assessment of hemoglobin levels at diagnosis and during follow-up may help inform risk stratification, particularly in higher-risk groups such as older adults, patients with chronic kidney disease, or those with recurrent hospitalizations. Identification of declining hemoglobin may also prompt evaluation for potentially reversible contributors, including iron deficiency, renal dysfunction, nutritional deficiency, inflammation, or occult blood loss.

Iron deficiency screening may be relevant because it is common and potentially modifiable, although direct evidence that correction of anemia improves hard clinical outcomes in HFpEF remains limited [28]. Accordingly, the present findings should be interpreted as supporting anemia assessment within comprehensive HFpEF management rather than establishing anemia status as a determinant of therapeutic selection. Further prospective studies and randomized trials are needed to determine whether targeted correction of anemia or related abnormalities can improve symptoms, hospitalization risk, or survival in HFpEF populations.

HFpEF may be particularly vulnerable to the effects of anemia because of its distinct pathophysiological substrate. Patients with HFpEF often exhibit ventricular-arterial uncoupling, increased myocardial stiffness, impaired relaxation, and substantial reliance on preload to maintain cardiac output. When hemoglobin levels fall, the resulting reduction in oxygen delivery and changes in vascular tone may precipitate symptomatic hypotension and tissue hypoxia in susceptible individuals. Furthermore, many HFpEF patients display chronotropic incompetence and limited ability to augment heart rate during exertion, potentially restricting adaptation to anemia-induced metabolic stress. Microvascular inflammation and endothelial dysfunction, central features of HFpEF, may further impair compensatory vasodilatory responses. Together, these factors provide a plausible explanation for why anemia may carry adverse prognostic implications in HFpEF, although direct comparative evidence with other heart failure phenotypes remains limited [29].

The present systematic review and meta-analysis have several notable strengths. It synthesizes data from a large and diverse HFpEF population across multiple geographic regions and clinical settings, enhancing the generalizability of the findings. Strict inclusion criteria were applied, focusing on studies with clearly defined HFpEF and anemia thresholds and reporting adjusted hazard ratios, thereby strengthening internal validity. The use of time-to-event estimates enabled a quantitative synthesis of prognostic risk, and sensitivity analyses were performed to assess the robustness of the pooled findings. Nevertheless, several limitations should be acknowledged, including substantial residual heterogeneity across studies, variability in HFpEF definitions (EF ≥40% vs. ≥50%), differences in follow-up duration, and the observational nature of most included cohorts, which may permit unmeasured confounding despite multivariable adjustment. In addition, the number of studies available for quantitative synthesis was small, precluding a reliable formal assessment of publication bias or small-study effects.

Future research should aim to prospectively evaluate the proposed “hemoglobin reserve threshold” hypothesis in HFpEF through dedicated cohort studies and randomized trials enriched for HFpEF-specific phenotypes. Stratification by renal function, inflammatory burden, frailty, and functional capacity may help identify subgroups most vulnerable to hemoglobin decline. Interventional studies specifically targeting anemia and iron correction strategies in HFpEF populations, rather than extrapolating from HFrEF trials, are particularly warranted. Such efforts could help clarify causality, define clinically meaningful hemoglobin targets, and inform precision-guided therapeutic approaches in this increasingly prevalent and heterogeneous heart failure phenotype.

## Conclusions

This systematic review and meta-analysis suggest that anemia may be associated with a higher risk of adverse outcomes, including all-cause mortality, in patients with heart failure with preserved ejection fraction (HFpEF). However, the magnitude of this association varied substantially across studies, and the certainty of evidence was very low, limited by the observational design of included cohorts, considerable heterogeneity, and imprecision. Accordingly, these findings should be interpreted as indicating that anemia may warrant consideration as a potential adverse prognostic marker, while current evidence cannot exclude residual confounding or differences in underlying disease severity. Although the available data do not establish a causal relationship, the observed association provides a rationale for systematic evaluation of hemoglobin status and potentially reversible contributing factors as part of comprehensive HFpEF management, pending interventional evidence. Current evidence does not establish that correction of anemia itself improves survival or reduces hospitalization, specifically in HFpEF. Further prospective studies are needed to clarify mechanisms, identify vulnerable subgroups, and determine whether targeted treatment strategies can improve clinical outcomes.
